# Contrasting Learning Psychology Theories Applied to the Teaching-Learning-Training Process of Tactics in Soccer

**DOI:** 10.3389/fpsyg.2021.637085

**Published:** 2021-05-04

**Authors:** Grégory Hallé Petiot, Rodrigo Aquino, Davi Correia da Silva, Daniel Vieira Barreira, Markus Raab

**Affiliations:** ^1^Department of Physical Education, Laval University, Quebec City, QC, Canada; ^2^Department of Sports, Center for Physical Education and Sports, Federal University of Espírito Santo, Vitória, Brazil; ^3^Post-Graduate Program in Exercise and Sport Sciences, Laboratory of Soccer Studies (LABESFUT), Rio de Janeiro State University, Rio de Janeiro, Brazil; ^4^Centro Universitário Governador Ozanam Coelho (UNIFAGOC), Ubá, Brazil; ^5^Center of Research, Training, Innovation and Intervention in Sport, Faculty of Sports, University of Porto, Porto, Portugal; ^6^Institute of Psychology, German Sport University Cologne, Cologne, Germany; ^7^School of Applied Sciences, London South Bank University, London, United Kingdom

**Keywords:** decision-making, team sports, soccer (football), pedagogy, epistemology

## Abstract

Research in sport pedagogy and its applied recommendations are still characterized by a contrast between the different learning theories from psychology. Traditional theories and their corresponding approaches to the specific case of teaching and learning “how to play [team sports like soccer]” are subject to compatibilities and incompatibilities. We discuss how behaviorism as an approach to teaching the game shows more incompatibilities with the nature of tactical actions when compared to constructivism. As coaches strive to teach the game and make their players and team perform, we argue that teaching the game requires teaching approaches that will help develop *their way to play* (i.e., tactical behavior) without taking away their autonomy and adaptiveness. The teaching-learning-training process for playing the game should then be conducted to harmonize the characteristics of the contents, the context, and the individual(s) at hand. We provide two illustrated examples and portray how the recommended approaches fit key contents of the game that are observed in the tactical behavior. We finally argue that the coherent design of games provides minimal conditions to teaching approaches, and that such a design should be a priority when elaborating the learning activities along the player development process. As a conclusion, the interactionist theory is the one that best serves the teaching of the game and the development of tactical behavior. We therefore defend that its principles can help coaches tailor their own strategy to teach the game with the many tools.

## Introduction

It is accepted that the development of athletes should occur in different degrees in each component such as the tactical, technical, physical, and psychological aspects of performance. Amongst them, tactics are essential to team performance because they take into account the context in which coordinated actions take place, and therefore influence other components of game play (e.g., technique) (Garganta, 1997). Such actions in the play are associated with perception, decision-making, and anticipation because they are considered as key elements to determine what to do in the play (Greco, 2006). Concretely, tactics are observed in the behavior of the player in terms of movements and positioning in relation to other players and the space/time (Teoldo et al., 2017). The evolution of this tactical behavior spans the whole long-term player development and will demand important efforts of coaching during learning phases since it is a source of influence for the way players perform in the play.

Players adjust the way they play by learning new skills and tactics and integrating the corresponding actions into their tactical behavior. In the context of team sports, actions are conceived as movements performed with an intention to reach an output (Samulski, 2009). A player executing a pass will therefore have a corresponding intention that is based on many possible notions of play, depending on the scope of stimuli that are considered, or the extent of his/her analysis of a situation in the play. For instance, a pass can be executed with the intention to maintain the possession of the ball in the team or to get rid of a group of opponents with the aim to progress toward their goal. In brief, intentions direct decision-making. They will manifest in the tactical behavior over the learning process and may refer to specific “contents of the game” that are assimilated as players develop their understanding of the game. The way players will acquire, assimilate, and integrate contents of the game firstly belongs to them even though it is indissociable from their teammates because they make sense of that content in their individual experience of the play. The ways these contents are taught should therefore be directed accordingly.

A clear framework based on robust learning theories and applicable approaches is important in the coaching of team sports due to the dynamic nature of tactics. Recently, approaches to teaching soccer have been classified and associated with theories of learning with the aim to clarify the conceptions and dynamics of acquisition in and for the context of play (Galatti et al., 2014). For instance, theories in learning psychology have long been able to govern the laws of simpler tasks. One of the traditional learning approaches named behaviorism helps “mastering early steps before progressing to more complex levels of performance” (Ertmer and Newby, 2013, p. 49) and thus fits the mechanist process of learning and rehearsing technical skills (Aquino et al., 2016). There is a significantly smaller knowledge base about the applicability of learning theories from psychology for open and complex activities than for closed-skills modalities (Gallahue et al., 2006), and this can make it difficult to help team sports players perform actions in the play. Another learning approach named constructivism can bring alternative solutions to the learning of actions in open skills like the ones utilized in the play (Gréhaigne and Godbout, 1995).

These two approaches, namely behaviorism and constructivism, are based on different conceptions of knowledge and on its acquisition (Driscoll, 2005). They are particularly relevant to the development of players because they influence the learning of the game in the context of competition, like in clubs and academies. A comparative analysis of the two approaches enlightens important principles that apply to these actions if focused to their applicability in the context of game play. Such analysis is therefore relevant to the challenges associated with teaching the game in team sports. Some of their principles could be useful to coaches if their applicability was revisited and analyzed in the context of play, starting with the understanding of their respective implications on the learning of the game. Coaches could be tooled up to skillfully build their own strategy to teach the game if they took into account the principles of learning actions for and in the play.

The aim of this paper was to analyze the applicability of classic learning psychology theories to the learning of actions in the context of play. More specifically, we analyzed tactics as the behaviors expressed by players in the specific context of play, throughout their development process. After reading this paper, practitioners should have a clearer idea of the applicability of the approaches (i.e., behaviorism, constructivism) to teach team sports with the intention to influence tactical behavior. We depicted the characteristics of the learning approaches and analyzed their commonalities with the interactionist theory to teaching the game. We also presented two practical examples that illustrate the (in)compatibilities between each approach and selected tactical contents of the game. Our proposal assumed that the long-term development process puts players at the center of all activities and leads them to become autonomous and resourceful (i.e., using principles of constructivism). The coach’s strategy to teach is meant to work as long as they are consistent with the specific demands from the context of play, the particularities of the tasks, and the actual individual development needs. This analysis was made with the aim to help coaches conduct long-term player development.

## The Development of Tactical Behavior

We chose the notion of tactical behavior as a concept that helps appreciate the individual “way of playing.” We interpret it as the most representative outcome of development in the long term because it reflects the applied result of learning the game. It embeds all the actions from passing the ball to moving into space, and all their adjustments made to play better throughout long-term player development. More specifically, tactical behavior depicts the individual patterns of movement and positioning for every player as well as the way they manage the space/time of play when involved in the play and in the organization of a group of players (Teoldo et al., 2017).

There is also a consensus that team sports feature dynamic relations of interdependence between the players in every situation-problem (Araujo et al., 2006). More specifically, team sports such as soccer are characterized by a relation of opposition and cooperation between the players in the play (Kannekens et al., 2009) in situations that researchers refer to as situations-problems (Teoldo et al., 2017). Teams organize to solve these situations-problems (Mombaerts, 1999) made of configurations, namely the grouping of teammates and opponents in an area of the field at a certain moment in the game (e.g., 3 v 2) (Caty et al., 2007). Players take advantage of possibilities that emerge in the play and that they can share with teammates (e.g., a pass that goes through a group of opponents) to operate their solutions (Silva et al., 2013). From an individual perspective, every tactical action on and off the ball should performed with the aim to create and seize these opportunities, to organize and to solve the situations-problems, under the constraints of time that are imposed by the context of play (Boulogne, 1972; Garganta et al., 2013).

A player’s tactical behavior portrays his/her way of playing as s/he will be better at performing actions over others, solving situations-problems better than others, and adhering to a certain degree to organization in the moment. It can be evaluated in respect of the efficiency and effectiveness of their action and of the result of these actions in situations of play (Teoldo et al., 2017). The tactical behavior emerges both (1) throughout the repeated experience of situations-problems in the play from early age levels and (2) the variety of these situations, and can change through time and experience (Teoldo et al., 2011). Players are in fact naturally developing their tactical behavior as soon as they play the game. They will, however, perfect it according to the diversity of situations that are proposed to them and their ability to perform the repeated and adapted actions. This is how players develop the experience, the knowledge, and the skills (e.g., perception of the information, judgment) that serve decision-making. Thus, the more the situations-problems are adjusted in adequacy with the needs and competency of the players as well as the representativeness of the context, the better the play could become (Pinder et al., 2011; Ertmer and Newby, 2013).

If tactical behavior can be used as an appreciation of the effectiveness of the response (i.e., actual executed solutions) to situations-problems or to the play, it should be tracked throughout the career of the players. Consequently, the approaches to developing tactical behavior during the formative process should be based on the evolution of the responses to the play. Following the recommendations in the scientific literature, these approaches should be adapted to evidence highlighted in the associated areas of investigation. Important considerations that were highlighted include findings on cognition (Casanova et al., 2009) and small-sided and conditioned games (Clemente et al., 2020). Such evidence notably supports adapted approaches to teaching in a long-term non-linear learning process (Práxedes et al., 2018), putting players at the center of teaching, training, and preparing for competition. As the coaches’ involvement in this formative process is judged as an important contribution, they are the ones who would benefit from the recommendations relating to the approaches to teach the game and their principles.

## Applicability of Learning Approaches to the Learning of “Actions in the Play”

To play means performing the movements as much as giving them a purpose to solve the situations of play throughout the game. Therefore, learning to play requires adopting approaches that will serve all components of the game. This reflects one of the most important challenges in coaching team sports as young players need to learn everything at the same time. The two aforementioned approaches to teaching denote (in)compatibilities with the needs of learning actions and consequently the approaches to the players that can be adopted to teach them. Table 1 explores the characteristics of behaviorism versus the ones of constructivism. Due to their differences, behaviorism and constructivism can be seen as two complete opposite ends since their foundations give the individual different roles. Their respective purpose, advantages, and limits to learning actions in the play can be interpreted if reviewed with the perspective of compatibility with the context of play.

**TABLE 1:** Characteristics of two opposing approaches to teaching according to Driscoll (2005) and Roberts and Potrac (2014).

	Behaviorism	Constructivism
Initial assumption	Knowledge exists independently and reflects the reality. It must be transferred from outside to within the learner	Knowledge exists through the experience of the learners and does not necessarily faithfully reflect the reality
Aimed output and time scale	Getting players to rapidly behave in *accepted ways.* Turn performance into habits and automatisms	Leading players to behave effectively and become autonomous with longer-term experience
Medium	Conditioners: praise and rewards reinforce right actions while consequences reinforce wrong ones	Proposing problems and interfering in the action with questioning and problems: prompts guide thinking for solution
Dynamic of learning	Repeat to assimilate a response	Test to construct a response
Teaching points	Direct instructions	Feedback and guiding for reflection
Evaluation	Final behavior is the main data	Judgment in decisions reflects the understanding of the game
Risks	Players respond to the conditioners rather than to content: players may expect the teacher to provide them with all the answers	Verbal answers to questions let learners believe they understand; integration into behavior still requires adequate practicing
Limits	A – Negative consequences are problematic when the player was not taught the new content or when the problem was not experienced before B – No dialogue, entire power and focus to the coach	A – Purely relies on knowledge of the game, can go in many directions B – Essentially needs time, autonomy, and adaptability as opposed to straightforward instructions
Reported applicability in sports	Execution of closed skills	Directing the understanding of the activity

Behaviorism is a classical conception of learning that is based on the belief that knowledge is objective and that must be transferred in the learner (Driscoll, 2005). It is therefore used as an approach toward the learner that uses positive and negative feedback and/or consequences as an instrument to create behavioral patterns that can become automatic if repeated (Ertmer and Newby, 2013). The behaviorist will prefer to keep the ownership and control of the content he/she wants to put in place, which practically limits the learner (the player) to the execution of predetermined actions. In line with this strategy, their approach to teaching amounts to behaviorism and practically focuses on conditioning performance and creating habits. This is the case for analytical technicist methods that aim to improve motor development and technical skills (Aquino et al., 2016).

On its end, constructivism rather encourages the construction of knowledge, following the assumption that knowledge exists through the individual experience (Driscoll, 2005). Hence, the knowledge does not have to be transferred; learning sports according to constructivism rather puts forward the adjustments of their own understanding and representation of the game. In its associated activities, learners reflect and create their own view of new experiences and conclusions by testing hypotheses and making sense of the result (Gréhaigne and Godbout, 1995). Such dynamic learning favors the understanding of the game because of the involvement of the individual in its thinking.

The contrast between the two opposite approaches is reflected in the conceptual gap about learning. This gap could be filled with another approach widely adopted in sport called cognitivism. For analysis purposes, we located it as one possible in-between proposal amongst approaches defined in the literature because it has specific commonalities with both ends. Cognitivism as an approach in teaching consists in instilling behaviors under control but features an active learning and processing of the information to assimilate knowledge (Driscoll, 2005). More specifically, it bids on the changes in the knowledge beforehand, which requires a highly demanding mental activity, especially for memory because the knowledge has to align with an external demand or guiding (Ertmer and Newby, 2013). This aims the instructions, corrections, and information to emphasize the natural cues from the environment that reflect the objective information but that cannot alone lead to learning to play. From that point, the team sports player is already given more responsibility and autonomy in choosing what to do in the play when using cognitivism compared to behaviorism but still features control over the action, this time similar to behaviorism.

Three alternatives from a preliminary network of approaches^1^ are offering their respective solutions to teaching actions in sports. As shown in Table 2, behaviorism and cognitivism are both considered to articulate objective knowledge (Driscoll, 2005). On the other hand, only cognitivism and constructivism are recognized as interactionist approaches since they are inherently conducted within the context of play (Scaglia et al., 2014). These two approaches reflect the conditions for the learners to reach their potential in relation to their autonomy in reading the play and making decisions (Scaglia et al., 2021). We also understand that, even if only these two approaches are showing a greater applicability to the play – at the expense of behaviorism – constructivism is more relevant to the need of building competencies for autonomy. As detailed in the next sub-sections, constant change in the environment of play needs specific characteristics in the knowledge of the game, the decisional process, and the capabilities favoring the non-linear learning of the game.

**TABLE 2:** Categorization of selected approaches based on Driscoll (2005) and Scaglia et al. (2014).

**Objective**		**Subjective**
Behaviorism	Cognitivism	Constructivism
	
**Empiricist**	**Interactionist**	

### Knowledge of the Game

Gréhaigne and Godbout (1995) insist that tactical knowledge, often at the source of efficient decisions in the play, is constructed through the repeated experience of the play and is not necessarily conscious (Beilock et al., 2003; Memmert and Roth, 2007). That knowledge is therefore of subjective and implicit in nature (Gallego et al., 2010; Mouchet, 2013) and is integrated and used in a way that allows players to judge the information they perceive as much as it helps them create a motor response to execute their decision (Petiot et al., 2017, 2021). Moreover, it is also argued that players are mostly able to learn new content if they can make sense of it because it is significant to them (Gallego et al., 2010; Mouchet, 2013). The association between new content and previous experiences is capable of motivating the player to explore, fail, and improve (Woods et al., 2020). The characteristics of the knowledge utilized in decision-making thus impose room for interpretation of reality as well as subjectivity. Such “owned” knowledge will emerge through the experience of favoring conditions of play and opportunities of the game, which will practically help players construct it and to review its content.

Above the individual perspective, players also need to adhere to the organization of the team, often referred to as “animation” in the technical jargon (Roberts and Potrac, 2014). More specifically, the overall team performance depends on the quality of interacting team processes, such as cohesion, shared and complementary mental models, and coordination (Silva et al., 2016; Santos et al., 2018). Teams with higher levels of social and task cohesion are more likely to develop shared (communal) and complementary (each players’ idiosyncratic knowledge) mental models which, in turn, allows teammates to be coordinated in space and time (Filho, 2019). In other words, the development of shared and complementary knowledge (or mental models) allows team members to do the right thing (know-what) at the right time (know-when) and for the right reason (know-why) (Filho and Tenenbaum, 2020). Every collective action is an emergent state, insofar that it reflects the integration of the actions of all the players in a situation of play.

### Decisional Process

It is acknowledged that the understanding of the game is of a different nature compared to ball mastery (Gréhaigne, 2014). The situations-problems that players are confronted with inside the play solicit skills including decision-making (Williams et al., 2006). Not only does the response to situations-problems differ all the time, decision-making in the context of play is highly constrained by time (Araújo, 2005; Mouchet, 2012). The functioning of the decisional process has therefore to adjust in order to efficiently work in the time that is given and lead to the execution of the chosen action (Petiot et al., 2021). More specifically, the decisional process is obliged to make major adjustments for cognition, resulting in heuristics described as essentially frugal and limited in information (Marasso et al., 2014; Raab, 2014).

Concretely, better decision-making skills are observed in players with more experience as they make more accurate and/or faster decisions than less experienced players (Giacomini et al., 2011). Players with more knowledge are making less cognitive effort to make their decisions (Cardoso et al., 2019, 2021) and employ cognitive mechanisms that allow them to make coherent judgment (Petiot et al., 2017) and still rely on their intuition (Raab and Johnson, 2008). Players showing a better tactical performance and a better knowledge of the game are therefore more susceptible to learning new things through the game (Greco, 2004; Iglesias et al., 2005). In sum, their ability to make better decisions also helps their ability to cumulate new knowledge that helps them play better (Giacomini et al., 2011).

It has also been shown that players who play in an organized team are required to manage two fonts of information in parallel, although events in the play prevails over other information that has been communicated by the coach prior to performance (Ribeiro et al., 2019). Even if both fonts of information help players self-organize and co-adapt to perform together in the play, processing all information may result in slowing down the course of action or holding back the decisional process, especially in the learning stages (Sohn et al., 2005). Practically, all the information that is shared prior to performance can be difficult for players to integrate whilst playing (Raab and Gigerenzer, 2015) and cause problems to the immediacy of decision-making in the play (Petiot et al., 2021). Even if it is still discussed how rich tactical decisions can be, literature presents them as highly cognitive since they embody content such as intentions (Samulski, 2009), perceptions (Roca et al., 2013), and judgment (Raab et al., 2019). We defend that these two fonts of information (i.e., stimuli in the play and directives from the coach) grow together in a reciprocal relation throughout the learning of the game, as long as the learning process accommodates making decisions and learning from them.

Furthermore, decision-making can be conceived as a core process within playing although it may not be exclusively cognitive. Cognition, behavior, and affective states might be intertwined in a three-way fashion, as detailed elsewhere (i.e., cognitive-affective-behavioral linkage; Tenenbaum et al., 2013), and akin to the notion of *reciprocal determinism* in applied psychology (see Tenenbaum and Filho, 2016). Put plainly, changing an athlete’s behaviors might change the way s/he thinks and feels; conversely changing the way an athlete thinks might change how s/he behaves and feels; likewise, an affective intervention (e.g., emotional intelligence training) might improve the behavior and cognitive functioning of the player and a given team as a whole even prior to teaching him/her content. The functioning of decision-making in the context of play also requires the experience of favoring learning conditions and interventions to adapt to the context, to the tasks, and ultimately to the behavior.

### Capabilities Favoring the Non-linear Learning of the Game

The characteristics that will make players integrate new content into their “way to play” include awareness and reading of the game (Scaglia et al., 2021). Such characteristics help players recognize the situations of play where the new content, intentions, or advice from the coaches apply. These skills and elements of information make the nature of learning essentially non-linear because it is not regulated by an established, step-by-step process (Chow et al., 2015). Instead, learning is based on individual experiences throughout a career, for which the player needs capabilities and abilities that serve the assimilation of new content. Amongst them, the principles of play are defined to give players the needed flexibility in their play without decreasing performance (Teoldo et al., 2017).

Principles are shown to require a more abstract understanding of the game that should emerge at appropriate stages in the development of players, either according to average cognitive capabilities associated with biological growth (e.g., Piaget’s stages of cognitive development) or individual development (e.g., Vygotsky’s zones of proximal development) (Piaget, 1964; Barrouillet, 2015). Abstract thinking, speculating about hypothetical situations, mental imaging, and deductive reasoning are all elements that observe substantial improvements during adolescence (Piaget, 1964). These mental abilities are directly associated with tactical knowledge and consequently with decision-making skills because of the need to create a representation of reality and testing actions. Correspondingly, it was suggested that the development of cognitive capabilities that help understand the core principles of the game of soccer appear around the age of 11, which is interpreted as an average to begin exploring the relation between players and their impact on tactical actions (Teoldo et al., 2017).

More characteristics associated with good decision-makers such as mental toughness and emotional intelligence (Fallon et al., 2014; Cowden, 2016) can also be interpreted as assets to integrate new content of the play. They add to the many capabilities and qualities that favor performance in changing environments as is the case for context of play. These individualities are developed when experiencing situations that will challenge and enrich them, hence the non-linear development of players (Ribeiro et al., 2021). Correspondingly, the conditions throughout the development process should be designed so that these specific capabilities flourish.

## A Matter of Harmonization Between the Conditions, the Content, and the Approach

It is the harmonization of the approach toward the player with the content to learn (ideas of the play) and the conditions (exercise-game) that will help the player/team learn and perform in the context of play. Such harmonization is convenient for the teaching-learning-training process that occurs through the regular formative activities in player development.

### Harmonization Between Conditions and Contents of Play

The idea of “action” should be inherently associated with the context of play as well as appreciated in more complete (and complex) situations. To be consequent to the particularities of decision-making in the context of play, actions in that context must be explored featuring a minimal complexity from early stages of learning (Memmert and Roth, 2007). This minimal complexity will help players insert their actions in the play even if they do not know how to do them yet (Araujo et al., 2009). Learning to play should therefore be conducted within the play as opposed to following dynamics that are based on static or pre-established stimuli, responses, and consequences (Davids et al., 2008). Learning the game will nevertheless benefit from delimited doses of the play in the form of content and conditions.

Players cannot be held exclusively accountable for the assimilation of new content, regardless if he/she is passive or proactive in the learning process. In fact, the context fulfills an important role in that process as it defines whether the player can afford to perform in the specific context of play given his/her competencies. Not only does the game in itself shape the way players decide in the play but it also conditions how they learn to play and how they behave in a response to new situations. Extensively, providing situations of play that will offer the right opportunities for the right content is therefore a prerequisite to learning, even before teaching. Such initial conditions will indeed favor learning as it emphasizes content although this context alone will not make learning automatic; the player will still have to challenge his/her skills to learn new contents.

In that effort, simple adapted repetitions of the situations of play are conceived as the activities that help players learn both the execution and the meaning of the actions, simultaneously, in the context that preserves the nature of decision-making described earlier. More specifically, it was highlighted in the literature that repetitions will differentiate through play even though the aimed actions still can be taught, learned, and trained through the process of “repeating [situations] without repeating [exactly the same actions]” (Garganta et al., 2013).

More specifically, practical experience of tailored situations of play will help players make sense of this new situation. Such experiences will solicit the tactical knowledge and skills associated with decision-making and require the players to adjust it to solve the situation-problem. Practically, the opportunity to effectively transfer learnings to actions will mainly depend on the actual opportunities arising in the play. Under inappropriate conditions of play, it is likely that players will be confronted with the obligation to deliver immediate and *efficient* outcome in the play (Musculus et al., 2019) and limit themselves to actions that can be judged as safe, but not aligned with the content to learn. In that case, players will hardly explore new solutions and consequently not learn new content of the game.

### Harmonization for Learning and Teaching the Game

The approaches to “learning how to play through games” have emphasized the interaction between the context and the players through adaptations in the structure of the play. Studies on small-sided games (SSGs) have shown that the design of games is made by shaping the possibilities that emerge in the play. Changing parameters (e.g., number of players) in the design of the exercise games will influence the tactical behavior as players should adjust their response to the situations that emerge with this new design. For instance, a game could be designed so that players strike on goals or change them to end-zones where players have to pass the ball. These designs can make it more or less complex and/or difficult to perform in that exercise-game and help players depending on the needs of learning (Machado et al., 2020a,b). In addition to the importance of *coherent* conditions, the teaching approach should emphasize that the lessons that have to be pulled out of the play from the situations-problems will help the player take ownership of the content that can help him/herself make better decisions.

The activity and teaching should also complement each other since the minimal conditions provided in the play will facilitate teaching and inversely, teaching will help players take advantage of the opportunities emerging in the play – that is to create solutions to the situations-problems. Situations and interventions should first make sure that the interaction between the information that comes from “outside” and “inside” the play (Ribeiro et al., 2019) observes a natural distribution so that it prevents the excess of information that comes from the coach (e.g., too many triggers). Tactical behavior will accomplish this from the repeated experience of tailored situations and their progressive insertion in team performance. With time, the more autonomous the players become, the more the teaching interventions can be adapted to leave players to come up with their own solutions, as divergent and creative as they can be, and let them explore possibilities and solutions in the play (Woods et al., 2020). In line with this premise, teaching has all the benefits in seeking a mutual consistency between the task inserted in the context of play and the intervention to instruct that task. Such dynamics of acquisition are only integrated in the approaches associated with the interactionist theory of learning (e.g., cognitivism and constructivism amongst other approaches).

In addition to playing tailored exercise-games, reviewing activities like questioning and/or other thinking exercises are also suggested to help the construction of knowledge, and thus can be considered as key teaching tools. These teaching tools are an important addition to the favorable conditions to learn and perform new content. Coaches should make abundant use of these tools, akin to a painter’s palette, depending on the need of learning. Only, no predefined combination is recognized as applicable in every situation: the duty to design and intervene for the right content, all this in harmonization, belongs to the coach. The challenge to direct the teaching-learning-training process of the game precisely lies in seeking this harmonization throughout longer-term player development. Yet, the interactionist theory and its associated approaches constitute a compatible framework to operate the combinations.

For the last few decades, many methodologies like Teaching Games for Understanding (TGFU) have led the practical area of teaching within games following holistic strategies (Gréhaigne et al., 2005; Raab, 2007). Despite nuances in their respective protocols, most of these methodologies feature tailored exercise-games as one main instrument to put potential problems in evidence, which has been proven efficient and massively reported in the context of teaching team sports for a *well-played game* (Fernández-Espínola et al., 2020). Hybrid methodologies like the SMART-ER model (Raab, 2015) seek to combine constraint-led exercises (e.g., SSGs) to thinking activities (e.g., debates) in a same methodology, assuming that it can reach more players at a moment or another. As long as all activities are adapted to an average level between the players, these combinations have the potential to help them progress individually in the long term because it will help cover determined tactical content and favor compatibility with the levels of performance or growth. To harmonize the interventions with these combinations, however, it is important to comprehend the (dis)advantages of the approaches to teaching.

## Applied Examples

For the purpose of showing how behaviorism, cognitivism, and constructivism harmonize or not with contents of the game, we have selected two common scenarios observable in careers, especially during player development. Both scenarios are challenging in the way they can be tackled in as much as how important they are in the development of the player’s tactical behavior. The proposed solutions reflect the importance given to the context of play as a context for learning counting with the additional activities/interventions. More importantly, they also align with the characteristics of the three approaches referred to earlier. They are, however, not compared to pedagogical methodologies.

### First Scenario: Breaking Habits With the Design of Game-Based Exercises

**FIGURE 1:**
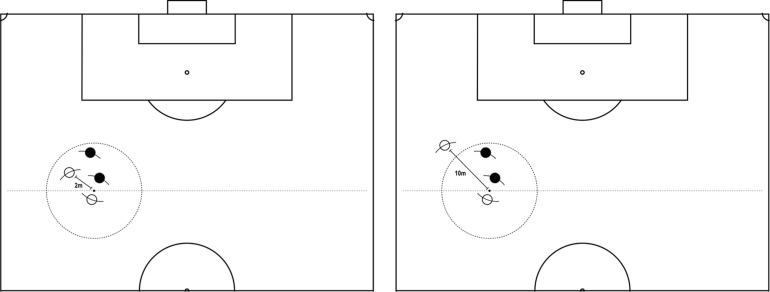
Comparison between creating an option of passing from 2 to 10 m.

It is common for clubs to recruit players who show great talent but were not trained in a formal structure from an early stage, and as a result show more difficulty in organizing with the rest of the team than with their skills. These incoming players denote great potential although their tactical behavior could be readjusted to address habits developed in the informal play they have been practicing on their own. This scenario can be the case for a player who has mostly played mini-games in tight spaces (e.g., some variants of street football) and will hardly receive passes more than two meters away from the ball carrier (Figure 1).

In such a case, talented players probably excel at keeping the ball for themselves in very tight spaces and close opposition. As a result, they may succeed for themselves but still lack the ability to manage the space of play and efficiently help their team to stay organized. Asking for the ball closer to the player in possession has become a habit to the detriment of collective tactics and may cause problems in the team. The player would benefit from proposing an option of passing from a longer distance to create space. More specifically, staying wide and deeper down the pitch helps decrease pressure on the carrier of the ball as well as the density of players in a given space of play. We assume that, in this scenario, the player would have to develop new competencies. On the other hand, the coach should help the player forge a new behavior by promoting strong tendencies such as, in occurrence, asking for the ball close to the player in possession. Habits in performing tactical action off the ball like the one in this scenario are particularly difficult to break.

It has been shown that changes in habits like this one must occur in the action by implicit learning (Lopes et al., 2018), or as mentioned earlier, repeating [the situation] without repeating [the same action]. If the player fails in applying tactical principles in his/her movement (e.g., receiving a pass from 10 m and controlling the ball), a successful teaching strategy is to emphasize a situation-problem within a tailored exercise-game. This exercise would consist of working with the player in potential situations-problems featuring a few other teammates, a tailored design of the space of play, and specific rules.

The proposed activity would therefore be utilized to condition the concerned player to respond to the rules that practically requires them to read the situation, judge, and test solutions. Intervention could then consist of rehearsing key principles of play repeatedly. The primary rules of this tailored exercise-game would persuade the player to explore the desired actions, or he/she would not be granted a point. Fewer but well-formulated rules would offer fewer alternatives to succeed. The more he/she succeeds in the desired outcome, the more the rules could be adapted to offer alternatives whilst encouraging the player to judge. With even more success, the rules can progressively be adapted to make it more difficult to judge the situation or execute that action although the instruction should still insist on trying and adapting.

As explored earlier, the suggested strategy to teach such content emphasized the situation-problem and added interventions to direct the response to that situation. Focusing on one specific action and omitting being clear on the rules that govern that exercise-game would lead to delays or mistakes in the play. If harmonized, this approach shows the potential to develop the corresponding competencies to read, judge, and decide with the aim to integrate adjusted actions in the tactical behavior. Additionally, it can help the player fit the team and subscribe to its way of organizing.

A typically behaviorist approach to correct actions in similar scenarios would employ rules specifically made for that player that oblige him/her to perform prescribed actions prior to receiving the ball or he/she will answer a conditioner (e.g., negative consequences if not performing that action). Using these conditioners, the player would have to perform the action numerous times in an extensive period of time to effectively embed the action in his/her adjusted tactical behavior, or he/she would return to old habits. Adopting behaviorism would thus be opposite to the idea of making the players autonomous because players would answer to the coach. The objective in helping this player should rather be to make him/her judge when he/she should perform such a movement as opposed to simply rehearsing an instructed movement off the ball. It would also be preferable not to condition the player with negative consequences but to encourage solutions that fit a desired response or even better, a principle. Even if the action was considered simpler and therefore approachable with conditioners, behaviorism still leads to more limits than benefits in the long term.

### Second Scenario: Creating Chances to Score a Goal

When putting in place a game model, coaches will seek to distribute roles and make the play flow. Some demands will require the involvement of more players than in scenario 1 to respond to key moments of the play. In such moments, all of the selected players should feel involved, *a priori* because they understand the shared, collective intention in the solution that is suggested to them. Individual actions to understand, assimilate, and integrate in the tactical behavior would result from the subscription to this shared intention.

**FIGURE 2:**
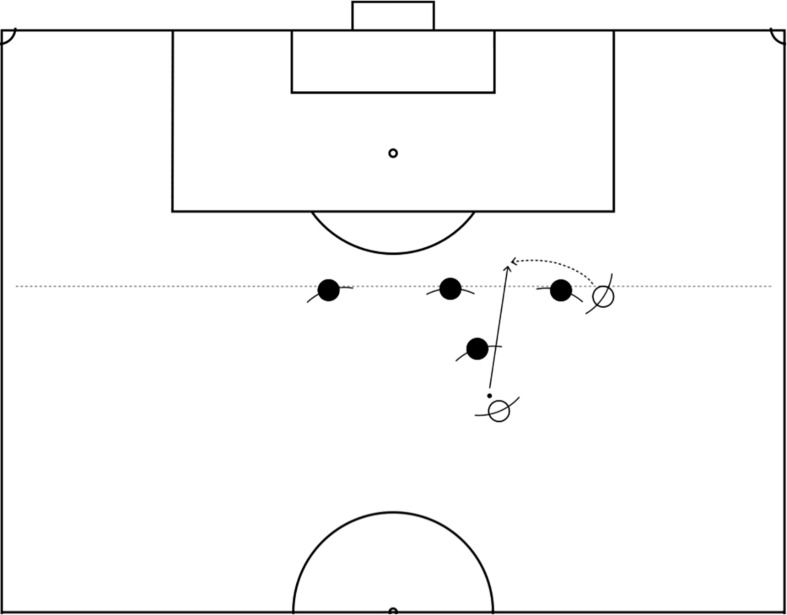
Seeking for the pass in the back of the defense line.

An action like the one aiming to run behind the defense line is rather initially appreciated in the play when the situation resembles an attack in a formal game (Figure 2). It is characterized by a great synchronization and use of space, probably not after the first pass. The players will have to read the play, recognize compatible situations, and seize the opportunity when it comes. They would then benefit from being patient and performing many principles of play to collectively create opportunities to rehearse the solution. For all these reasons, it would be essential for the players to perform the solution and all the actions that precede it. In this scenario, the core tactical principle “depth mobility” as initially defined by Bayer (1995) could easily be taken as a reference as it corresponds to the action of running with the specific intention to reach a space behind the defense line. As defined in the most recent work on the topic, the principle of depth mobility precisely consists in performing movements in the back of the last defense line, with the aim to amplify the effective space of play (da Costa et al., 2009). This principle can be used to direct the shared intention in the players involved in situations-problems and seize the corresponding opportunity.

In contrast with the first scenario, learning how to put in place the run behind the back of the defense line inherently relies on learning why it is necessary to organize and perform actions in a specific way. It will also take time to integrate all actions in a continuous play as the participants will have to recognize the opportunity when it comes without necessarily emphasizing it as is the case in the training exercise of scenario 1. For this reason, it is important to continue providing the players with many opportunities to “repeat without repeating” the actions and adapt them to the content of play. Coaches would then need to re-adjust the rules and parameters to meet the more natural context of play without decreasing the clarity of the opportunities nor of the objective. In that sense, it is crucial that coaches create the necessary minimal game conditions for the opportunities to arise “clearly” so that all players and the coaches themselves emphasize the actions to perform. Shaping conditions, that is determining the rules and parameters of the game, is key to the emergence of the corresponding opportunities that will help the players perform the aimed tactical content.

Thus, the needs of learning that particular content, the characteristics of that specific action, and the context in which it is inserted create altogether a *more* constructivist approach. With the repetition of the situation and additional thinking activities, the players would then be guided to the conclusion that the aimed run is the action to perform to successfully respond to the situation-problem in respect to the game model. While the first step remains to show and emphasize the situation-problem on the field, the tools for teaching include key additional thinking activities that all aim to understand the play. In such a context, questioning players is key to gathering them in a common, shared understanding of the play and resulting roles in the organization of the team.

The aimed outcome in similar scenarios should be directed by the interaction and mutual relationship between the coach, players, and context of play. On one hand, it would be expected that the player responds to the trigger recommended by the coach but, on the other hand, it would still be assumed that he/she would create his/her own model of the situation. In this regard, investigations on decision-making have highlighted the subjective nature of decision-making and its usefulness even to improve decisions in team sports (Mouchet, 2013; Gesbert et al., 2017). Making players experience the problem so that lessons and training become significant and representative to them can help build their own knowledge around the specific aimed content to learn. More specifically, such knowledge would embed the understanding of the game and the representation of the play that will help them recognize and solve situation-problems.

In occurrence, constructivism is capable of guiding these contextualized lessons and enlighten important options when they occur in the play. Teaching interventions that correspond to constructivism can be considered as an additional guide that can help players respond to the situations-problems. Such guiding should for instance consist of emphasizing the pre-objectives and objectives to reach (i.e., dislodge the opponent, receive passes away from the opponents) (Travassos et al., 2012). Reminding players of the objective can redirect the intention of the actions without prescribing and teaching pre-objectives can help players create the conditions needed to execute the aimed actions without actually instructing them.

The two examples illustrated in this section were designed to help understand the compatibilities between content of the game and each aforementioned approach to teach the game. The result of the analysis enlightens key strategies to teaching the game that we judge useful to coaches over long-term player development. The fact that there are different needs in the long-term development process and even in a single week of training reinforces the need of harmonizing the approach toward players with the content and the context (i.e., the design of the exercise) in different activities. In that sense, the teaching-learning-training process should be seen as a dynamic combination of activities and interventions aiming to help achieve development objectives and maintaining the levels of performance over time. Coaches may be more impactful in maintaining efficiency in the tactical behavior of the players if making the necessary adjustments to make sure the harmonization between the context and the content stays alive. Similarly, building their teaching strategy toward players in respect to the levels of complexity and difficulty of the tactical content they want to get across as well as the competency of their players will help them address the issues to solve in the tactical behavior of players.

## Considering the Complexity, Difficulty, and Competency

Players will respond differently to the conditions of the play, notably the complexity and the difficulty of the situations of play. A higher degree of cognitive activity often caused by complexity can be observed in potential delays in execution or impulsive responses to the play. A balanced proposition of new content will help players perform without suffering delays for extended period of time whilst learning (for more information about “reinvestment,” see Masters and Maxwell, 2004). On the contrary, conditions help maintain short timescale decisions, the objective being to help cognitive information promote the decisional process and to keep the flow of actions in the play whilst maintaining their quality (Petiot et al., 2021). For this reason, activities have to be chosen adequately and the demands from the coach have to fit the actual competencies of the players. More precisely, approaches to teaching must, however, be consistent with the capabilities of the individual to receive new content of the game, learn and integrate it into their own play, and perform it under pressure.

This can be addressed by modifying parameters of the exercise-games to decrease/increase the number of possibilities in the play and the pressure of time and/or space in the play. That would have an influence on the complexity and difficulty of the game, like for instance obliging players to judge the best opportunity in the moment of the game and increase the speed of execution of their actions (Sgrò et al., 2018). Despite a clearer understanding of complexity and difficulty of the game, there is still no evidence-based indication of the exact levels of complexity and difficulty that players need to experience according to level of competency/and indirectly age, nor how to put in place the measured levels in the play throughout the career of the players.

In a key study on games and their parameters, it was shown that the play varies in complexity according to the volume of information that is considered when making decisions (Machado et al., 2019a). The quantity of information more frequently originates from the number of players interacting in a given situation of play. The levels of complexity of a game can be calculated in comparison to the formal competitive game of soccer (Travassos, 2014, p. 79). This helps appreciate how complex a situation can become.

$$Complexity\left(\%\right)=\frac{\begin{array}{c} \mathrm{number}\mathrm{of}\mathrm{opponents}\mathrm{in}\mathrm{the}\mathrm{task}*\mathrm{number}\mathrm{of}\mathrm{action}\\ \mathrm{possibilities}\mathrm{of}\mathrm{player}\mathrm{with}\mathrm{ball}\mathrm{possession}\mathrm{in}\mathrm{the}{\text{task}}^{\text{2}} \end{array}}{\begin{array}{c} \mathrm{number}\mathrm{of}\mathrm{opponents}\mathrm{during}\mathrm{the}\mathrm{official}\mathrm{match}*\\ \mathrm{number}\mathrm{of}\mathrm{action}\mathrm{possibilities}\mathrm{of}\mathrm{player}\mathrm{with}\mathrm{ball}\\ \mathrm{possession}\mathrm{during}\mathrm{the}\mathrm{official}\mathrm{match} \end{array}}\times 100$$

Hence, the number of players and targets reflect the quantity of possible opportunities of actions that can be explored, even though they will all depend on the perceived, processed information that is used to make decisions in situations of play. The more information there is, the more complex the situations of play (i.e., the problem to solve) can become. On top of this, types of targets or other rules that can be applied to a game also influence decision-making and the response to the situations.

Another similar research has also shown that the situations of play will vary in levels of difficulty, which refers to the number of free possibilities that players can transform in actual action (Machado et al., 2019b). The difficulty can also be calculated as a ratio of the formal game (Travassos, 2014, p. 78). Similar to complexity, this can help appreciate how difficult it can become for players to find and execute solutions to situations-problems.

$$Difficulty\left(\%\right)=\frac{\mathrm{number}\mathrm{of}\mathrm{opponents}\mathrm{in}\mathrm{the}\mathrm{task}}{\begin{array}{c} \mathrm{number}\mathrm{of}\mathrm{action}\mathrm{possibilities}\mathrm{of}\mathrm{player}\mathrm{with}\mathrm{ball}\\ \mathrm{possession}\mathrm{in}\mathrm{the}{\text{task}}^{\text{2}} \end{array}}\times 100$$

The fewer possibilities, the more difficult it gets for players to perform actions that will directly help their team reach an objective (e.g., score a point; see Machado et al., 2019b). Together with complexity, the difficulty of a game will have an influence on the response to its situations-problems (Machado et al., 2020b). Naturally, the more competent players are, the better they should manage the situations and provide an advantage to their team.

Exercise-games featuring four or five players in each team (“4v4s” and “5v5s”) will provide easier possibilities to perform some collective tactical principles than “3v3s” (Clemente et al., 2020). Thus, in the context of play, complexity and difficulty are intertwined (Machado et al., 2019a). According to findings in cognitive sciences, the complexity of the game requires processing (filtering/discriminating information, judging) whereas difficulty will challenge the use of skills (Weinberg and Gould, 2014). As learning players are facing new content in both technical and tactical aspects at the same time, they will first need exercises that offer a balance between these contents since they will likely not be able to perform all demands when required. For this reason, there is an advantage to plan back and forth between less complex and difficult to more complex and difficult situations along the learning phase, especially for exercises dedicated to content of the game. As suggested in scenario 1, simpler exercise-games can serve to necessarily decrease the complexity and difficulty to enlighten situations-problems and guide decisions. Such emphasis will also help coaches run the thinking activities according to the response from the players.

## Building a Strategy to Teach the Game

In the realm of youth team sports, coaches may be reported adopting strategies to teach positioning and movements that are similar to the ones utilized to teach ball mastery or general technique. Increasing evidence in the literature about learning styles also supports that some players will use the help of one approach more than others (Fuelscher et al., 2012) and benefit from clear, direct instructions for positioning to perform in the short term. In a very practical perspective in youth competitive sports, the decision of the approach toward the players lies in putting a team in place to win the next game versus developing smart players in the longer term. Such a dilemma can be felt every week throughout a season. Under the pressure of immediate results, coaches may resort to fixing short-term problems – the most urgent needs – to keep working on other improvements thereafter. There is still a need to balance processes between preparation and development to play well even if being pressured with the importance of results.

In such a context, the study of the theories of learning and of their associated approaches toward the players can answer long-term objectives recommended in curriculums for player development. As a starter, it can be observed that methodologies to teach the game proposed in recent publications in scientific literature (Raab, 2007, 2015) tend to integrate activities designed with constraints to answer the challenges raised through the non-linear learning process (Davids et al., 2008; Serra-Olivares et al., 2015). In fact, the organization of these teaching methodologies seem to be converging toward the assembling of exercises based and on well-designed situations of play with key reviewing activities, depending on the levels of complexity, difficulty, and competency. They can thus be compared to cognitivism and constructivism for their emphasis on the constant interaction between individuals and the context of play.

These approaches to direct the teaching-learning-training process are thus compatible with this idea of assembling exercise-games and interventions precisely in the way they are utilized for teaching, learning, and/or training. Game-based activities are reported to be utilized to teach, to learn, or to train, separately (Scaglia, 2020; Scaglia et al., 2021). In occurrence, in formal practice environments, the context of play is generally used as an instrument of training and/or of teaching with the aim to reach higher levels of understanding, performance, and potentially results, notably through the orientations given from the coach. Cognitivism and constructivism can notably satisfy the needs of learning in a context of competition and highly demanding training activities like is the case in soccer academies. Cognitivism, however, features more directiveness from the coach. Constructivism, on its end, is meant to be more beneficial for the individual but should be efficient as long as it is harmonized in terms of conditions and contents of the game. In that sense, its adoption requires coaches to make clear for themselves what they want to achieve with their players and the interaction they want to add over the experience of the play itself. They will also have a greater impact on players if the coaches make a good reading of these characteristics, their combination in interaction, and their evolution over time.

As deducted from the analysis proposed in this article, coaches should value constructivism and its associated holistic variants under the umbrella of the interactionist theory (e.g., ecological, socio-cultural) and refrain from adapting prescriptive behaviorism for the development of tactical behavior even if it can look more efficient to correct actions or prepare a team to perform. In fact, behaviorism would not contribute to the development of full potential or adaptive play (Scaglia et al., 2021). In fact, behaviorism imposes its own range of applicability and should be judged as incompatible to the development of tactical behavior. A minimal interaction and complexity are needed in the solving of problems in the play and should be embedded in the activities proposed for the development of tactical behavior, most of the time. Otherwise, tactics and decisions are neutralized, and their ownership is denaturalized.

**FIGURE 3:**
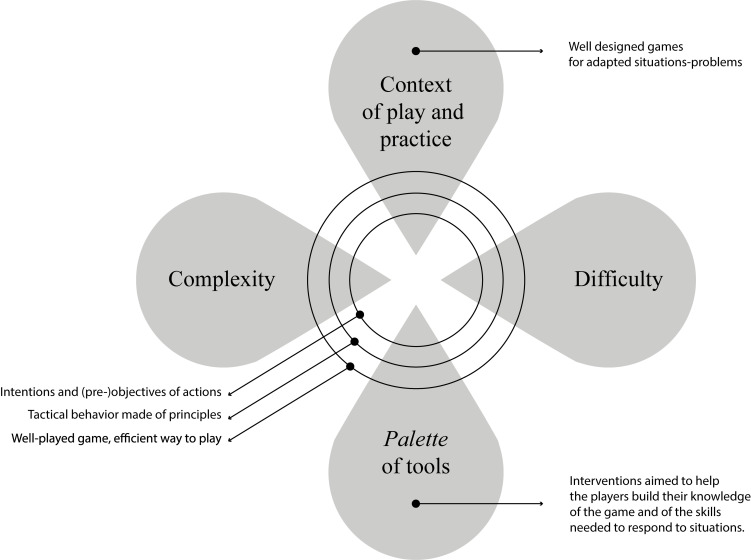
Interpretation of the assembling of the main elements useful to interactionist theories to teach and learn the play (e.g., Constructivist, Ecological and Socio-Cultural approaches).

As illustrated in Figure 3, key activities and interventions subscribe to Gréhaigne’s (2011) recommendation to use many tools to help players learn and corresponds at many levels to constructivism. More specifically, integrating debates, questioning, and feedback integrated to well-designed exercise-games can help players make adjustments to improve decision-making – that is the *what to do* in the play. Practically, teaching the game should be tailor-made for players and the content that is being covered during a training session, even if game-based activities can create more tasks for coaches (Clemente, 2016). They can help answer the individual needs of learning and respond well to a wide span of combinations between individuals, contexts, and tasks utilizing a palette of tools, as opposed to adopting one single approach for all needs. An accurate reading of the characteristics of the individual, the task, and the context will help coaches propose the right exercise and approach them adequately.

The massive volume of information that comes from these combinations must be integrated alongside the process with the sole objective to help players develop their potential even if it spans a longer development period. Developing a way to play – that is a tactical behavior – should thus be seen as ongoing “smooth changes” in players’ decisions and actions in the play. The higher the level, the more players will be required to be have an efficient tactical behavior. After all, coaches should seek to make players as adaptive and experienced as possible with the aim to prepare them for the next level of competition. Players will also have to develop key competencies (e.g., creativity) in order to be resourceful in responding to specific situations of play (Petiot et al., 2020). This is reflected for instance in finding solutions to situations-problems subscribing to a model as much as performing disruptive solutions for the same principle. Approaches associated with interactionism (e.g., constructivism, ecological, socio-cultural) thus fit the needs to instill such autonomy in players as opposed to teaching with the aim to assimilate all solutions, *drilling* a player with a rich environmental process.

We thus support that the adoption of approaches associated with interactionism to player development will have a positive and significant influence on players’ tactical behavior and on their “way of playing” as a whole. With constructivism as a model, the interactionist family of approaches is the one that best articulates the dynamics of learning that are relevant and compatible to playing and to learning to play. They will also give coaches the flexibility to read and judge the combinations of context, content, and tasks, and make adjustments along the way. They also give room for tools and being adaptable to teaching, learning, and training for the game. As an initial step in the exploration of these approaches to teaching, understanding how the underlying implications of these approaches toward players can help tailor adequate learning contexts and teaching interventions. Further, reflecting why the adoption of an approach went well or wrong can help coaches discern their applicability and reach objectives.

## Implication for Directing the Teaching-Learning-Training Process and Conclusion

The characteristics of learning theories and approaches analyzed in this article can be used as a guide to direct the teaching-learning-training process effectively. Coaches should be aware of their applicability since their initial theoretical assumption, their conception, and their functioning are fundamentally different. They will have a different impact on the tactical behavior of developing players in the short and long term and will have an influence mainly on their autonomy and adaptiveness. Their adoption to teach, train, or prepare players to respond to specific situations-problems will also influence the interventions toward players. In that sense, some interventions will be more convenient for the experience of the players.

Our analysis was precisely made to support the search for more convenience in the development of players in terms of their tactical behavior in the play, for the specific demands of the context of play. The examples were illustrated with the intention to contextualize the use of approaches to teaching the game and intervene in the tactical behavior of learning for players. Both scenarios reflect common challenges that coaches can be confronted with and they can be tackled using approaches associated with interactionism for teaching the game (e.g., constructivism, ecological, socio-cultural). We discussed that the specificity of the context of play practically imposes their corresponding dynamics of learning and therefore cannot benefit the learning of actions in the play because of the implications on decision-making and response in the play. It remains clear that the use of the adapted context of play is an important ingredient to developing actions and decision-making as changing parameters create conditions to achieve or challenge the performance of tactical actions. These conditions are important for the emergence of opportunities players should recognize, take advantage of, and practice through repetition within the variability of situations-problems. They are also ideal initial conditions to actual teaching interventions like providing feedback and asking questions that are relevant to the situation-problem at hand. The repeated experience of the adjusted play, adequate interventions, and the competition are the elements of the practice that lead to an adjusted tactical behavior, and therefore what portrays the long-term teaching-learning-training process.

Coaches would therefore benefit from being aware of their style as well as their predispositions to adopt certain approaches over others. They should specifically consider their pedagogical sensibility, their intentions, the structure of training sessions, and more importantly the players’ intrinsic motivations in their strategy to teach the game because it influences the teaching and learning as well as the relationship with the player (Lafrenière et al., 2011). It is important for coaching staff to assess coaching behavior on the field with the use of assessment grids (Guzmán and Calpe-Gómez, 2012) although such an assessment helps pinpoint the influence coaches have on the experience of the players whilst participating in activities (Cushion et al., 2012). For a better understanding of pedagogy, assessments should inform the approaches that were effectively adopted and if the intervention corresponded to the aimed objective. Such feedback has the power to track and help balance the adoption of the approaches to teaching the game depending on the individuals and the content. Such tracking would specially be useful when coaches expect certain responses or when the content has already been covered but it is not observed in the play because it would inform them how to make adjustments.

This article, its recommendations, and its conclusions are limited to the scope of tactics as opposed to a model that embeds multiple facets of development or education. We understand that the development of tactics is one of many parts of player development, especially if conceived as part of their overall sportive education. Using the framework of tactics to instigate an appropriate approach to teaching actions for the play still can be considered as relevant due to the dominance of tactics in team sports (Teoldo et al., 2017). The context of play in itself integrates most of the demands and stimuli to conduct the development of the action for the play, and for this reason, can be used as a satisfactory framework for development. We also understand that player development cannot be limited to the examples proposed in this article, neither should the teaching-learning-training process be limited to the concepts of tactics. Similarly, the concepts presented in relation to tactics are adapted to the game of soccer and should observe adjustments when applying to other sports, even in the family of team or invasion sports (Thorpe et al., 1986).

## Author Contributions

GH and RA were responsible of elaborating the initial topic. DS contributed with the content associated to tactical behavior. DB contributed with the content associated to the components of the play and their influence on actions in the play and vice-versa. MR contributed to reviewing the overall coherence in the references to theories and concepts. All authors contributed to the article and approved the submitted version.

## Conflict of Interest

The authors declare that the research was conducted in the absence of any commercial or financial relationships that could be construed as a potential conflict of interest.

## Footnotes

1 We purposefully limited this preliminary analysis to the approaches listed in the table as a means of highlighting their commonalities and differences and do not neglect the existence of other approaches.
